# Emphasising Personal Investment Effects Weight Loss and Hedonic Thoughts about Food after Obesity Surgery

**DOI:** 10.1155/2014/810374

**Published:** 2014-06-02

**Authors:** Margaret Husted, Jane Ogden

**Affiliations:** Psychology Department, FAHS, University of Surrey, Stag Hill, Guildford, Surrey GU2 7XH, UK

## Abstract

Obesity surgery is the most effective treatment method for the severely obese but does not work for everyone. Indications are that weight-loss success may be related to individuals' sense of investment in surgery, with failure linked to higher automatic hedonic motivations to consume food and greater susceptibility to food in the environment. A pilot study using an independent experimental design recruited bariatric surgery patients (*n* = 91) via a UK obesity-surgery charity website who were randomly allocated to either the intervention or the control condition. The intervention involved raising the salience of the personal investment made in having weight-loss surgery in an attempt to reduce automatic hedonic thoughts about food and aid weight loss. Data was collected initially with subsequent weight loss measured at 3 months of follow-up. Following the intervention, participants reported significantly reduced hedonic thoughts, increased liking for low-fat foods, reduced liking of high-fat food, and higher self-efficacy for achieving sustained weight loss than controls. By 3 months, this was translated into significant differences in mean weight losses of 6.77 kg for the intervention group and 0.91 kg for control participants. To conclude, a quick simple cost-effective intervention encouraging participants to focus on investment helped weight loss and changed hedonic thoughts about food in bariatric patients.

## 1. Introduction

Obesity surgery may be viewed as the last resort by patients, but it is the treatment of choice for the medical profession when dealing with the severely obese. The numbers of obese people doubled in the USA and tripled in Britain between 1980 and 2000 [1–4]. Obesity is established as having many significant negative health consequences. Recent estimates indicate that 5.5% of all cancers are attributable to being overweight with the relative risk of cancer increasing by 12% per BMI increase of 5 kg m^−2^ based on meta-analysis of studies [5, 6]. Weight-loss surgery is the most effective treatment for the severely obese in comparison with conventional treatment [7–9]. There are moderating factors when comparing weight-loss outcomes such as age, starting weight, quality of life, and surgery type [7, 10]. A large minority of patients do not experience successful outcomes, and repeat, revised, or secondary surgery can occur for 25–30% of patients [1, 11]. There is also evidence of weight regain, with 30% of individuals starting to regain weight 18 months after procedure [12]. A minority return to presurgery levels, but in general weight loss is still significantly greater than nonsurgical alternatives, with 16–30% of excess weight lost remaining after 10 years [1, 11, 13]. Whilst celebrating the successes, it is nonetheless clear that there is still a need to improve outcomes further.

Factors identified as playing a significant part in whether individuals weight-loss outcomes are a success or a failure include hedonic thoughts about food and the level of personal investment [14, 15]. A person's investment is the extent to which they have committed to a task and the effort that they have had to undertake, whether this is in terms of time, finance, emotion, or physical and behavioural effort. Research examining successful weight-loss maintenance shows significant differences in psychological characteristics and health behaviour between successful surgical and successful nonsurgical weight-loss participants [16]. Users of traditional methods of diet and exercise potentially work harder, requiring significant personal commitment of time and effort. For the successful ones, these kinds of behaviour persist over time. In contrast, surgical participants eat higher percentage fat diets, more fast food, and fewer breakfasts, have higher levels of depression and stress, and undertake significantly less exercise [16]. The depression and stress could signify cognitive dissonance caused by the conflict between their eating behaviour and their investment in weight loss through surgery. Some eating behaviour will be a direct result of surgery side effects, such as dumping and modified taste perception, but it is also a common response to hedonic thoughts about food. In a recent study, significant predictors of postoperative weight regain were increased food urges, decreased postoperative wellbeing, and additional addictive behaviour, but, in contrast, postoperative self-monitoring (a conscious investment in the task) was associated with weight-loss maintenance [17].

Studies of reward-related cognitive processes provide evidence that differences in hedonic motivation for food can influence uncontrolled and emotional eating behaviour [18–20]. Some individuals demonstrate higher wanting for high-calorie foods through increased brain activation and attention following food cues exposure and reduced satiety following food consumption [21, 22]. Neural studies indicate that hedonic responses to food seen in obesity are similar to those evident with drug use and other addictions [23].

Dual-processing theories propose individual behaviour results from motivation and attention being influenced by two cognitive processing paths: controlled and automatic. Cognitive Goal Theory [3] suggests that obesity be understood through the dual-processing framework and that unless individuals gain dominance over the quicker, automatic processing path, their natural tendency and response to food result in failed weight-loss attempts [24]. Support for dual-processing theory can be seen in computational and neural modelling [25, 26]. Although findings indicate that automatic processing generally dominates over controlled processing (particularly when under cognitive load), there is evidence that the transition between processing paths can be subject to an individual's control if learnt or through using adaptive strategies [26]. FMRI research indicates individual differences in the reward drive seen by enhanced food cue reactivity in obese individuals [21, 27, 28]. EEG evidence of where this enhanced reactivity occurs in the attention process or whether it is the same for all obese subjects is mixed [29, 30]. Acknowledging dual-processing theory, the experimental intervention proposes to aid the controlled process, the conscious weight-loss goal, remain dominant over the individual's automatic hedonic response by using personal investment in surgery.

Support for the use of personal investment is found in previous research. Qualitative research shows that successful dieters using obesity medication expressed greater adherence to dietary and behaviour change due to their increased investment as a result of the unpleasant drug side effects [31]. Dieters reported the experience of side effects as motivational to behaviour change as when they strayed from healthy eating there was a clear negative consequence, that is, sickness and anal discharge, which motivated them to try harder to maintain a healthy eating pattern. Some obesity surgery patients report less food preoccupation due to viewing the surgery as imposing control on them; as control is given voluntarily, individuals express more investment in outcomes [32]. Achievement of weight-loss maintenance appears influenced by the length and number of past weight-loss attempts [33]. This suggests a learning process occurring alongside significant personal investment. It is the combination of investment and learnt behaviour patterns which leads to successful behavioural change—behaviour moving from controlled to automatic. Successful dieters have been shown to consciously activate their dieting goal following exposure to food cues, whereas overweight unsuccessful dieters do not [34, 35]. Foods motivational pull reduces when individuals are primed with diet concepts as a form of enforced self-regulation [36]. The present study examines whether using investment as the priming concept is an effective alternative. The influence of hedonic thoughts over some people's cognitive processes arguably provides an explanation for why some surgical outcomes, as well as traditional treatments, are prone to failure. Using investment to moderate subsequent food thought is grounded in theories of cognitive dissonance [37]. To be rational, individuals try to ensure that their subsequent actions fit with previous cognitive choices; where they do not, cognitive dissonance arises [15, 38]. In this instance, once an individual has focused on the cost to them of the surgery, personally, financially, and socially, to then maintain a high cognitive attention and a food-reward view should cause dissonance.

To summarise, although obesity surgery has demonstrated clear effectiveness in weight loss, results are not the same for everyone and weight regain is still common. Considering dual-processing theory and evidence of increased hedonic motivation in obese individuals, the aim of this research is to test the effectiveness of an investment based intervention in reducing hedonic thoughts about food and see whether changes in cognitive response translate into subsequent behaviour. The hypothesis is that hedonic thoughts about food will be reduced for postsurgery participants following the intervention in comparison with the control group and that this will translate into improved weight loss by follow-up.

## 2. Method

### 2.1. Participants

91 participants took part in the study. Postsurgery participants were recruited from a specialist weight-loss surgery charity website. Basic demographic information of gender, age, height, and weight was requested, and individuals unable to provide this data were excluded from the study. No reimbursement was offered for participation. Ethical approval was obtained from the University of Surrey Ethics Committee.

### 2.2. Design

The study involved an experimental design with two conditions: control versus investment based intervention which was designed to raise the salience of the investment that the participant had made through their decision to have surgery in terms of financial, social, personal, and physical costs.

### 2.3. Measures

Participants completed measures relating to their demographics, hedonic thoughts about food, self-efficacy, and behavioural intentions at the initial time point, and weight change was taken at 3 months of follow-up. Participants reported age, gender, height, and weight and supplied surgery-specific information including type of weight-loss surgery, time since surgery, and weight currently and prior to surgery. Hedonic thoughts about food were measured in terms of emotional eating response, hedonic wanting, and liking.

#### 2.3.1. Emotional Eating

This was assessed using the 13-item emotional eating subscale from the Dutch Eating Behaviour Questionnaire (DEBQ-EES) [39]. The participants considered that the extent of their desire to eat had been influenced by emotion within the preceding 2 weeks. 89 participants fully completed the emotional eating measure. The scale adopted a 5-point range from 1 “not at all,” to 5 “very often.” A high score is indicative of emotion having a strong influence on participants eating behaviour. Studies show that the DEBQ-EES correlates highly with other recognised emotional eating scales (*r* = 0.80) and demonstrates high reliability and validity across populations [39, 40]. When scale reliability was assessed for the current participants, Cronbach's alpha was 0.94, with internal consistency indicated by interitem correlations of .61 to 0.84.

#### 2.3.2. Hedonic Wanting

We used the Power of Food Scale (PFS) [41] to measure hedonic wanting, and 86 participants provided complete responses. The scale measures appetite for palatable foods based on proximity, rather than consumption, the hedonic motivation aspects of food environment. The measure comprises 15 items with a 5-point response ranging from 1 “don't agree at all” to 5 “strongly agree.” A high score indicates greater motivational draw and increased wanting of food in response to cues in the environment, potentially influencing subsequent eating behaviour. The scale proposes to contain three factors: food available, food present, and food tasted, with the factor structure reproduced and scale validity demonstrated in subsequent research [42]. The PFS scale validity alpha is of .94. Internal consistency ranged from 0.52 to 0.80. Test-retest reliability has previously been shown: *r* = 0.77, *P* < 0.001 [41].

#### 2.3.3. Hedonic Liking

In relation to food palatability, preference was examined using written and visual representations of food types. Responses to this measure were received in full from 85 participants. This measure forms the second element in the distinction in hedonic hunger motivation suggested between reward liking (the taste component) and wanting (the drive to eat) [43]. 16 food items were divided into four groupings replicating measures suggested in previous studies [34, 44]. The four groups were the following: Low Fat Low Sugar; Low Fat High Sugar; High Fat Low Sugar; and High Fat High Sugar. Participants expressed their preference for the food type by rating the extent to which they liked or disliked the taste based on a 9-point hedonic scale, ranging from 1 “extremely dislike” to 9 “extremely like” [45].

#### 2.3.4. Other Measures

Self-efficacy and behavioural intentions were measured by participants rating their intentions to eat and self-efficacy at achieving weight loss using 5-point Likert scales ranging from 1 “not at all” to 5 “very much.” Subsequent weight change was measured at 3 months of follow-up with participants asked to provide their current weight.

### 2.4. Procedure

The research adopted an independent experimental design with participants providing questionnaire data at time one and self-reported weight change at time two. Participants accessed the questionnaire through a web-based link to a secure hosting site. After providing informed consent, participants completed the questionnaire which contained an embedded experimental intervention designed to raise their salience concerning investment. At this point, they were randomly allocated to either the control (*n* = 46) or the intervention (*n* = 45) conditions.

The experiment met a priori requirements to achieve acceptable statistical power [45, 46]. Computer randomisation of participants was used in order to allocate participants to conditions. The individuals in the intervention condition completed the investment section of the questionnaire prior to proceeding with the other measures. The control group did not undertake the investment task at any stage in order to try and identify if any short/medium term effect would be seen in subsequent weight loss.

### 2.5. The Investment Intervention

In order to raise the salience of the degree of investment participants had made in having obesity surgery, participants allocated to the intervention group (*n* = 45) were asked to complete a simple 12-item questionnaire about their experiences of surgery. This was developed specifically for the research study, and the items requested the individuals to consider the ways in which the surgery had impacted upon them financially, socially, personally, and physically. To this end, the questions and responses were framed in such a way as to emphasise investment with examples of items being the following: (i) financial, for example, “how much has the surgery impacted on you financially?”; (ii) social, for example, “to what extent has your decision to have surgery affected your immediate family?”; (iii) personal, for example, “how big a commitment do you feel you made to yourself by having weight-loss surgery?”; (iv) physically, for example, “how much general discomfort have you had following your surgery?”. The 5-point responses ranged from 1 “not at all” to 5 “very much.” The study met minimum requirements of participants to item ratios [47]. Preliminary investigations of the internal reliability of the investment scale are positive; Cronbach's alpha is .81; scale mean = 42.15; SD = 6.95; and item mean is 3.51.

### 2.6. Data Analysis

Analysis of demographic and clinical data was undertaken on the whole data set and by group. Initial analysis of variables ascertained distribution. Skewness and kurtosis were calculated and examined against a cut-off of ±1.96. Questionnaire variables, with the exception of “high-fat combined” (which encompassed participant liking responses to the High Fat Low Sugar and High Fat High Sugar foods), were normally distributed. Intervention effects were assessed by comparing differences between surgery participants in the intervention group to the control group in terms of their hedonic thoughts about food and subsequent weight loss at 3 months of follow-up. A range of tests were used as appropriate including* t*-tests, Mann-Whitney test, chi-square test, correlation analysis, and ANCOVA.

## 3. Results

Group characteristics are provided in Table 1. No systematic differences between the experimental conditions are apparent on age, BMI, gender, surgery type, time since surgery, or weight loss since surgery. The mean BMI of all participants based on self-report height and weight was 36.98 (SD = 11.41) with a range of 20.7–66.8. Participant mean age was 43.17 (SD = 10.09). Participants had undertaken surgery on average 23.83 months previously (SD = 28.14) with an average weight loss of 41.56 kg (SD = 27.93). The majority of participants had undertaken RNY gastric bypass surgery.

## 4. Impact of the Intervention

The immediate impact of the intervention on hedonic thoughts, self-efficacy, behavioural intentions, and subsequent 3-month weight loss is shown in Table 2.

The intervention group had significantly lower scores with respect to the Power of Food aggregate (*t*(84) = 1.61, *P* < 0.05) and PFS factor “food present” (*t*(84) = 2.10, *P* < 0.01). The intervention participants expressed a significantly greater preference for low-fat food (*t*(83) = −1.67, *P* < 0.05) and greater dislike of high-fat food (*U* = 667.0, *z* = −2.05,   *P* < 0.05, *r* = .22). The intervention group reported greater self-efficacy, with the intervention participants having significantly greater confidence in their ability to achieve sustained weight loss (*U* = 596.0, *z* = −2.80, *P* < 0.005, *r* = .30). Follow-up data at 3 months showed that weight change ranged from a loss of 20.41 kg to a gain of 9.07 kg. Group differences were significant. The intervention group had a mean additional weight loss of 6.77 kg versus the control group additional mean weight loss of 0.85 kg (*t*(28) = −2.44, *P* < 0.05). Weight change in participants was correlated with participants intention to eat less, *r*_*s*_.459, *P* ≤ 0.01, and confidence in achieving sustained weight loss, *r*_*s*_.385, *P* ≤ 0.05.

Due to the high attrition rate in this study in order to determine true effects of the intervention best practice recommends intention to treat (ITT) principles are adopted through the imputation of missing data [48]. We report both per protocol (PP) and intent to treat (ITT) results; however, subsequent interpretation is based on ITT results. The ANCOVA was undertaken to determine the effect of the intervention on subsequent weight change whilst controlling for variance in weight at time 1, time since surgery, and weight loss since surgery. The results show a significant effect of the intervention on weight change at 3 months: *F*(1,25) = 6.269,* P* = 0.01, and *η*_*p*_^2^.20 (PP) and *F*(1,89) = 5.992, *P* = 0.01, and *η*_*p*_^2^.06 (ITT). According to Cohen's [49] guidelines partial eta-squared (*η*_*p*_^2^) values of 0.01, 0.06, and 0.13 constitute small, medium, and large effect sizes, respectively; therefore, based on the more conservative ITT analysis, the effect size for the intervention was medium independent of covariates. The ITT analysis also indicated that weight loss since surgery also explained a significant amount of variance in the weight change at 3 months, *F*(1,89) = 6.063, *P* = 0.01*η*_*p*_^2^.06 (ITT). This indicates weight loss since surgery has a medium size effect on weight change in addition to the intervention effects and controlling for time since surgery and variance in weight at T1. 

Analysis of the relationship between the 3-month weight change and the individual intervention items showed significant correlations for two particular items: “how much has the surgery impacted on you financially?”, *r*_*s*_.563, *P* ≤ 0.05, and “how big a commitment do you feel you have made to yourself by having weight loss surgery?”, *r*_*s*_.578, *P* ≤ 0.05. In addition, 3-month weight change correlated with participants intention to eat less, *r*_*s*_.429, *P* ≤ 0.05, and self-efficacy in achieving sustained weight-loss, *r*_*s*_.375, *P* ≤ 0.05.

To control for that fact, there was a difference in distribution of surgery type between groups (albeit nonsignificant), and a comparison was made to ensure that effects were not the result of higher numbers of gastric bypass patients being in the follow-up intervention group. Results show that the intervention effect is still significant, with gastric bypass patients in the intervention group (mdn = 11.5) having significantly greater weight loss than gastric bypass patients in the control group (mdn = 6.36): *U* = 16.5, *z* = −1.99, *P* ≤ 0.05, and  *r* = −0.47.

## 5. Discussion

The present study explored the impact of investment on hedonic thoughts about food and weight loss after surgery. In terms of hedonic thoughts, the initial hypothesis was largely supported. The impetus behind the research was to investigate a way to reduce foods motivational draw through emphasising individuals' commitment to surgery with the hope that any positive cognitive change achieved aids future weight loss and maintenance, thus reducing surgery failures and weight regain. The results indicate that significant differences following the intervention were evident with effects continuing for the short-term resulting in significant differences in subsequent weight loss. Traditionally negative associations of surgery such as pain, discomfort, or financial costs are minimised after surgery, but this research indicates that the opposite strategy may be effective in achieving long-term weight loss and maintenance.

Although the intervention group participants expressed significantly greater levels of hunger, it is felt that this did not influence the results as the outcomes are counterintuitive to what you would expect if hunger itself was creating a motivational draw [50, 51]. The focus on the individuals' investment in the surgery and effort taken to lose weight may rejuvenate self-efficacy in achieving and maintaining their weight-loss goal and their intention to eat less. This is particularly relevant considering that self-efficacy appears as a predictor of health behaviour change outcomes and weight loss specifically [52, 53]. The relationship between self-efficacy and intention has long been established with recent research showing that it is when motivational self-efficacy combines with behavioural intention that behavioural outcomes in weight loss are positive [54].

The interventions focus on investment in the weight-loss surgery resulted in participants demonstrating lower cognitive responses to the power of food. It is proposed that when individuals are confronted with what they actively choose to do, in this instance undertake surgery, the motivational drive of food seen in hedonic thoughts and feelings of lack of personal control are diminished and this is then translated into effective weight loss. Past research indicates in normal circumstances that hedonic drive for food is stable [41] which supports the assumption that the intervention is influencing responses at a cognitive level. It is the case that responses could be influenced by social desirability effects; however, research indicates that social desirability has a detrimental effect on subsequent weight loss [55], and, therefore, if the change in hedonic thoughts was a result of social desirability bias rather than the intervention, you would expect the subsequent weight change for the intervention group to be reduced. The results suggest that individuals may be able to retrain their cognitive responses and reduce influences of hedonic thoughts by focusing on their investment in the weight loss task on a similar line to successful dieters' activation of their dieting goals [34–36].

The intervention, perhaps surprisingly, had a significant effect on participants' expressions of liking for food. Past research indicates that people's taste response, or liking, does not differ but that obese participants generally express greater motivational drive, or wanting, for high-fat foods [56, 57]. Recent studies have indicated that following bariatric surgery participants experience changes in neurological responses to food cues leading to a reduction in preference for high-fat foods [20, 58]. In contrast, with this study only intervention participants expressed significantly greater liking for low-fat foods and a greater dislike for high-fat foods than controls. As evidence indicates that alterations in taste perception after surgery are not the same for all and variations in food urges predictive of postoperative weight regain [17, 59], results are encouraging as they indicate that an individual may be able to affect their food preference using basic, timely interventions. It should be acknowledged that there is significant overlap in the definitions of hedonic liking and hedonic wanting [60]. Therefore, where it is not possible for participants to engage in food taste tests to determine liking ratings (as is the case with this study) the measures being used may be partly accessing the participants hedonic wanting for food, and not just their hedonic liking for it.

It is logical to anticipate that the further away from the surgery, the lower levels of confidence for some individuals, due to reductions in motivation to adhere with postsurgery requirements and so forth. This position is supported by weight-regain evidence [12]. It may therefore be appropriate to target postsurgery participants a number of months after surgery to combat reductions in self-efficacy and sustain motivation for maintenance of behaviour. The 3-month follow-up data shows a significant difference in weight-loss between experimental groups. On average, control participants lost a further 0.85 kg (2 lbs), whereas the intervention participants lost an average 6.77 kg (14.9 lbs), *P* ≤ 0.05. It is not clear how the cognitive changes following the intervention translated into behaviour as participants may either have altered their eating behaviour or alternatively adhered better to postsurgery recommendations.

It is speculated that the intervention makes use of dual-processing paths and enables the more conscious controlled processing, engaged with weight loss and dietary change, to remain dominant over the subconscious automatic process, the hedonic response. The results from the follow-up weight-loss data of the experimental participants indicate the possibility that the intervention effects continue, at least for the short term. As past evidence demonstrates that weight-loss surgery does not provide successful outcomes for all [11, 12], the results indicate that the use of investment to address part of the failure in weight-loss achievement and maintenance previously seen is effective.

## 6. Implications for Future Theory and Research

The results provide support for theories suggesting that cognitive responses to food can be altered in order to aid weight-loss and its maintenance, but further replication and exploration are necessary. Past research shows that successful dieters demonstrate the capacity to consciously activate positive healthy cognitive responses appropriate to their weight-loss goal [34–36]. In this case, the use of an investment appears successful in reducing hedonic food thoughts and maintaining more positive weight-loss cognitions. This is a conscious, effortful process, likely using a controlled processing path, as suggested by dual-processing models. When considering cognitive dissonance, where individuals look to minimise conflicting, irrational behaviour, this also requires the active engagement of controlled processing. The exact mechanisms and brain activation occurring cannot be established through studies such as this, but a similar experimental manipulation could be applied in the context of neurological research to try and establish further evidence of cognitive processing differences or activation and compare the effectiveness of different manipulations.

## 7. Methodological Limitations

With the study being reliant on participants from a typically clinical population, the sample size is restricted and further replication is required to substantiate results. The study is reliant on self-report of height and weight and it would be preferential to have been able to independently validate the weight information provided. The study purposefully adopts an intervention manipulation that was subtle in order to help reduce this limitation. The experiment met a priori power calculations but the participant numbers in follow-up were reduced, and, in order to determine a true intervention effect, imputation of missing data needed to be undertaken. Therefore, the preliminary power of results was reduced; however, a significant intervention result remained. A key limitation of the design, however, was that additional measures were not taken at time two to allow for analysis of whether the group differences in questionnaire responses were maintained. A more robust longitudinal design would allow exploration of causality and substantiate whether the changes in motivational response and hedonic thought are stable or transient. Although steps were taken to identify any systematic differences between groups or confounding variables influencing outcomes, it is still inappropriate to make any direct causal links from the research.

## 8. Conclusion

Weight-loss surgery offers substantial initial weight loss and is the most effective treatment method for severe obesity, but outcomes are not successful for everyone. There is a need for interventions to address the psychological aspects of eating behaviour that surgery alone does not fix and which potentially explain failures in weight-loss achievement and maintenance. This research demonstrates a simple intervention, focusing on individual's personal investment and effort taken in having surgery, positively affects individual responses to food and increased subsequent weight loss. There is a need to replicate findings and test the durability of any manipulation but, in the current environment where quick, cheap, easy, and accessible solutions are needed, these preliminary findings offer indications of the possibility to address cognitive responses to food in a positive manner and further improve surgical outcomes.

## Figures and Tables

**Table 1:** Group characteristics of experimental conditions.

Variable	Control (*n* = 46)	Investment (*n* = 45)	*t* or *χ*	*P*
	Mean (SD)	Mean (SD)		
Age	45.17 (9.50)	44.56 (8.68)	*t*(89) = 0.32	0.74
Gender	M = 4, F = 42	M = 4, F = 41	*χ*(1) = 0.001	0.97
BMI	36.85 (10.48)	35.39 (8.78)	*t*(89) = 0.71	0.47
Surgery type			*χ*(3) = 5.10	0.16
Gastric band	12	4		
RNY gastric bypass	26	33		
Duodenal switch	4	3		
Other	4	5		
Time since surgery (mths)	22.96 (29.88)	27.48 (27.70)	*t*(89) = −0.749	0.45
Weight loss after surgery (Kg)	36.84 (27.99)	47.33 (28.63)	*t*(89) = −1.76	0.08

Source: postsurgery participant data.

**Table 2:** Intervention effects on hedonic thoughts and weight loss.

Variable	Control (*N* = 46)	Investment (*N* = 45)	*t* or *χ*	*P*	Effect size *r*
	Mean (SD)	Mean (SD)			
Emotion	2.97 (0.97)	2.88 (1.11)	*t*(87) = 0.42	0.33	
PFS aggregate	3.24 (0.98)	2.88 (1.06)	*t*(84) = 1.61	0.05	0.17
PFS available	3.25 (1.03)	3.0 (1.14)	*t*(84) = 1.03	0.15	0.11
PFS present	3.40 (1.18)	2.86 (1.22)	*t*(84) = 2.10	0.01	0.22
PFS tasted	3.09 (1.06)	2.75 (1.08)	*t*(84) = 1.47	0.07	0.16
High-fat foods	58.35 (6.86)	52.57 (11.97)	*U* = 667.0, *z* = −2.05	0.02	0.22
Low-fat foods	48.12 (7.9)	51.24 (9.15)	*t*(83) = −1.67	0.05	0.18
Self-efficacy in future weight loss	3.10 (1.35)	3.91 (0.97)	*U* = 596.0, *z* = −2.80	**0.002**	**0.3**
Sensation to eat	1.85 (0.92)	1.82 (1.11)	*U* = 842.0, *z* = −0.552	0.29	
Hunger	2.15 (0.92)	2.51 (0.89)	*U* = 723.0, *z* = −1.64	0.05	0.17
Intent to eat less	3.78 (1.16)	4.04 (0.87)	*U* = 806.0, *z* = −0.870	0.19	
F/up weight change (kg)	−.85 (6.00)	−6.77 (7.21)	*t*(28) = −2.44	0.02	**0.4**

Source*:* postsurgery participant data; PFS: Power of Food Scale [40].

Cohen's effect size [49]: small 0.10; medium 0.30; large 0.50.
